# Plate Load Tests of Soft Foundations Reinforced by Soilbags with Solid Wastes for Wind Farms

**DOI:** 10.3390/ma16114173

**Published:** 2023-06-03

**Authors:** Chenchen Zhang, Jie Liao, Yuchi Zhang, Liujiang Wang

**Affiliations:** 1 College of Water Conservancy and Hydropower, Hohai University, Nanjing 210098, China; zhangcc1988@foxmail.com (C.Z.); ettaha@163.com (J.L.); 2 Department of Hydraulic Engineering, Zhejiang Tongji Vocational College of Science and Technology, Hangzhou 311231, China; zhangyuchizyc@gmail.com; 3 College of Civil Engineering, Sanjiang University, Nanjing 210012, China

**Keywords:** plate load test, sustainable construction material, soilbags, solid waste, soft foundation, wind farm

## Abstract

Soilbags are expandable three-dimensional geosynthetic bags made from high-density polyethylene or polypropylene. This study conducted a series of plate load tests to explore the bearing capacity of soft foundations reinforced by soilbags filled with solid wastes based on an onshore wind farm project in China. The effect of contained material on the bearing capacity of the soilbag-reinforced foundation was investigated during the field tests. The experimental studies indicated that soilbag reinforcement with reused solid wastes could substantially improve the bearing capacity of soft foundations under vertical loading conditions. Solid wastes like excavated soil or brick slag residues were found to be suitable as contained material, and the soilbags with plain soil mixed with brick slag had higher bearing capacity than those with pure plain soil. The earth pressure analysis indicated that stress diffusion occurred through the soilbag layers to reduce the load transferred to the underlying soft soil. The stress diffusion angle of soilbag reinforcement obtained from the tests was approximately 38°. In addition, combining soilbag reinforcement with bottom sludge permeable treatment was an effective foundation reinforcement method, which required fewer soilbag layers due to its relatively high permeability. Furthermore, soilbags are considered sustainable construction materials with advantages such as high construction efficiency, low cost, easy reclamation and environmental friendliness while making full use of local solid wastes.

## 1. Introduction

Wind power is a crucial and promising source of clean and renewable energy, with enormous potential for growth both commercially and technically [1]. In recent decades, the utilization of wind energy has increased significantly worldwide [2]. Wind power generation is the main force of green energy as it plays an important role in reducing greenhouse gas emissions, thereby attenuating global warming [3]. The future direction of wind power generation lies in the development of high-rise towers in plain areas and offshore wind power [4]. However, the construction of hoisting platforms on soft foundations poses a significant challenge for wind power projects in plain areas [5]. The lifting equipment required for wind turbine generator units with high lifting heights and large lifting capacities is super large tonnage cranes, with crawler cranes above 600 t commonly used. The key to lifting operations using such heavy equipment involves providing a hoisting platform with adequate bearing capacity. Since the hoisting platform is a temporary operating platform, its foundation needs to be restored to its original appearance after construction. The reclamation, construction progress and overall cost are all key factors to be considered. The conventional approach of replacing soft foundations with granular materials such as sand or building waste brick slag [6,7] proves inefficient and expensive, not conducive to overall progress and cost control. Therefore, there is a pressing need to develop a more sustainable and efficient construction material or technology for addressing the challenges posed by deep soft foundations in plain wind farms.

Soilbags are expandable three-dimensional (3D) geosynthetic bags made from high-density polyethylene (PE) or polypropylene (PP) and filled with earth-rock materials, which have been used for flood control and emergency rescue or some temporary retaining structures for a long time [8]. In recent years, extensive and systematic research on soilbags [9,10,11] has been undertaken to explore their engineering characteristics, uncovering numerous advantages. Studies conducted by Mastsuoka [12], Liu [13], Shen [14] and Wang [15] have demonstrated that vertically piled soilbags can effectively reduce mechanical vibration through experimental investigations and DEM analysis. Li [16,17] has conducted a number of tests that reveal that soilbags exhibit less frost heave and thawing settlement compared to soils under similar conditions. Martinelli [18] and Recio [19] have explored that sandbag barriers may stabilize the positions of natural sandy bars and coastal structures. Jia [20] found that the increase and distribution of the tensile force in the bag material have an important effect on the compressive strength of soilbags. Due to these attributes, soilbags have been gradually used as permanent or semi-permanent materials in building foundations [21], retaining walls [22,23], subgrades [24,25], coastal groynes [26], channel slopes [27,28], and other civil and hydraulic structures.

Bearing capacity and settlement issues associated with soft soil foundations are major concerns for engineers and researchers engaged in geotechnical and civil engineering projects [29]. Research over the past few decades has consistently demonstrated that geosynthetic reinforcement presents an effective solution for enabling structural construction on soft soil foundations [30,31]. Among various geosynthetic materials, soilbags have emerged as a highly effective and sustainable foundation treatment technology due to their ability to enhance bearing capacity [32]. Matsuoka’s findings [33] indicated that soilbags could increase the bearing capacity of soft ground by 5–10 times due to their high compressive strength. Xu [34] has carried out plate load tests on foundations reinforced by soilbags containing sand and found that the ultimate bearing capacity for cases without soilbags, with two layers of soilbags, and with three layers of soilbags are 70 kPa, 160 kPa, and 240 kPa, respectively. Bai’s study [35] discussed the loading deformation characteristics of marl foundations with large and small soilbags based on in-situ load tests. Hataf [36] found that the number and arrangement of soilbags are the most important factors in increasing bearing capacity and decreasing foundation settlements. However, the studies mentioned above have primarily focused on using coarse granular materials or low-cohesion soils such as sands and gravels as infill materials for soilbags. These materials are always lacking at construction sites and need to be transported from elsewhere. Recent research by Wang [29] found that soilbag reinforcement with reused excavated soft soils as the contained material can substantially improve the bearing capacity of the soft foundation, indicating that excavated solid waste can be used as soilbag filling material. Nevertheless, the bearing capacity of foundations reinforced by soilbags containing excavated solid waste has rarely been studied.

This paper investigates the feasibility of using soilbags filled with excavated solid wastes for reinforcing the soft foundation of hoisting platforms in a plain wind farm project in China. Specifically, plate load tests are conducted to assess the bearing capacity of soft foundations reinforced by soilbags. The study examines the effectiveness of soilbag-reinforced foundations along with a comparison of the reinforcement effects of two types of solid waste materials contained in soilbags. In addition, the study investigates the effect of stress diffusion between and under the bag layers. Furthermore, the study explores the influence of water permeability on soilbag reinforcement by examining the effectiveness of combining soilbag reinforcement with bottom sludge permeable treatment.

## 2. Materials and Methods

### 2.1. Project Background

**Figure 1:**
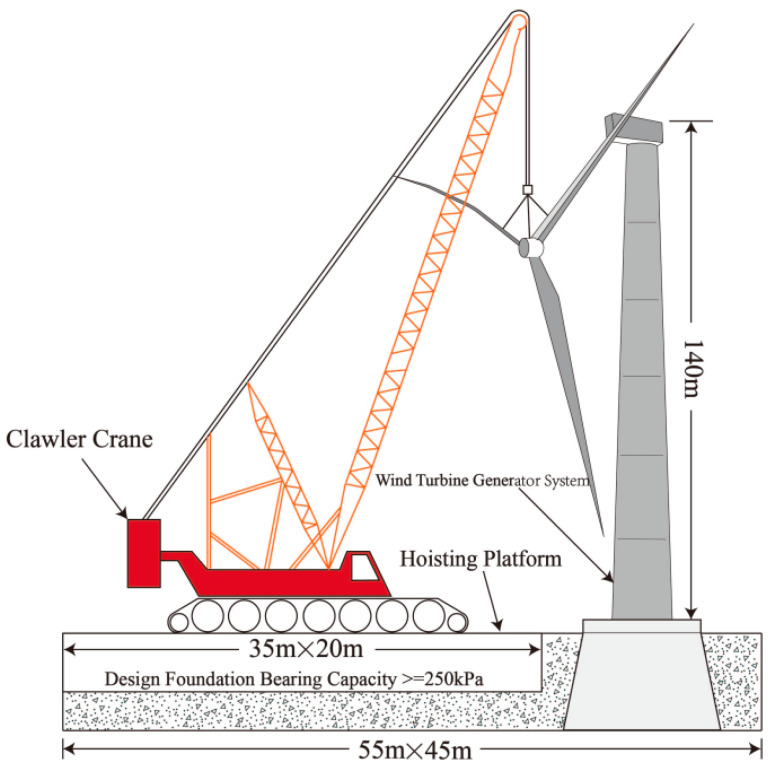
Schematic diagram of main crane operation area.

The field tests are based on a wind power construction project located in the northern Jiangsu Plain, with a planned total installed capacity of 97.5 MW and 39 sets of 2.5 MW piece-type mixed tower wind turbine generators. The mixed tower wind turbine foundation consists of a pile foundation and cavity-bearing platform connected to the upper tower by 12 bundles of anchor cables. The wind turbine tower comprises 4 sections of mixed towers and 3 sections of steel towers. The height of the fan wheel center is 140 m, and the impeller diameter is 146 m. The maximum lifting weight of a single section of a mixed tower is 291 t. The size of the main crane hoisting platform of this project is 20 m × 35 m. It is proposed to use the Zoomlion ZCC9800z crane as the main hoisting machine, and the schematic diagram of the main crane operation area is presented in Figure 1.

Furthermore, the average pressure on the foundation of the hoisting platform when the crane is lifting can be calculated according to the equipment parameters, performance and hoisting technical scheme [37]. According to the wind farm design report, the foundation bearing capacity calculated for the main crane operation area is required to be not less than 250 kPa. The ultimate bearing capacity of the foundation *f_u_* is calculated by multiplying the characteristic value of the foundation bearing capacity *f_ak_* by the safety factor *f_s_*, which is 2.0 [38]. Hence, the ultimate foundation bearing capacity of the main crane hoisting platform here must meet *f_u_* ≥ 500 kPa.

The landform of the wind farm area belongs to the Lixia River shallow depression plain, which is generally flat and open. The geological surface layer of the project area is plain fill soil or sludge soil, below which are plastic-hard plastic clay, silty clay and slit. Thus, the bearing capacity of the foundations is quite low. Most of the fan sites are located in the lake deposition or beside the river ditch, with a thick sludge layer that may be more than 6 m deep. In order to meet the foundation bearing capacity requirements of the construction hoisting platform site, the original construction method is to replace and fill, which is to excavate the original soft layer and sludge soil, then to replace and fill with construction waste such as sand or brick slag compacted in layers, and to lay a steel plate interlayer at the same time. It is relatively simple to use this method for construction sites with thin, soft soil layers. However, if the soft soil layer is thick, the amount of replacement and filling will be enormous, resulting in low construction efficiency and high costs. Furthermore, due to the large number of fan sites in the project and the synchronous construction of multiple sites, the consumption of brick slag is large. Nevertheless, the brick slag and other construction waste in the surrounding market cannot meet the construction demand, and the construction progress is also affected by the backfill material source. For this reason, soilbag technology is proposed to reinforce the hoisting platform foundations in this project to reduce the amount of brick slag, improve the bearing capacity of the foundation, and meet the hoisting construction requirements on-site.

### 2.2. Technical Principle of Soilbags

Soilbags are expandable geosynthetic bags made by putting soil and rock materials into woven bags with certain specifications and material characteristics. According to the research [39], the soilbag is compressed and deformed after being subjected to external force, which causes the bag to stretch. Thereafter, a tension *T* is generated in the bag. At the same time, the tension *T* will restrain the soil in the bag, which in return, increases the contact force between the soil particles. Meanwhile, the shear strength of the soil in the bag increases. Hence, soilbags can greatly improve the bearing capacity of the foundation as a reinforcement layer arranged in the soil layer close to the foundation. According to the Mohr–Coulomb strength failure criterion, the relationship between large and small principal stresses of soil in bags under the limit state can be expressed as [9]

(1) $$\sigma_{1f}=\sigma_{3f}K_{p}+2c\sqrt{K_{p}}+\left[2T\left(\frac{1}{H}+\frac{1}{L}\right)K_{p}-2T\left(\frac{1}{B}+\frac{1}{L}\right)\right]=\sigma_{3f}K_{p}+2\left(c+c_{T}\right)\sqrt{K_{p}}$$

(2) $$c_{T}=\frac{T}{\sqrt{K_{p}}}\left[\left(\frac{1}{H}+\frac{1}{L}\right)K_{p}-T\left(\frac{1}{B}+\frac{1}{L}\right)\right]$$

where *σ*_1*f*_ and *σ*_3*f*_ are external loads; *c* is the cohesion of soil inside the soilbag; *φ* is the internal friction angle of soil; *T* is the bag tension; *B*, *L* and *H* are the length, width and height of soilbags, respectively; *K*_p_ = (1 + sinφ)/(1 − sinφ), which is equivalent to the passive earth pressure coefficient of the soil in the bag; *c_T_* is the additional cohesion caused by soilbag tension.

It can be observed from Formula (2) that the additional cohesion *c_T_* caused by the tension of soilbags is the combined result of the comprehensive effect of the soil strength in the bag, the bag tensile force *T* and the shape of the bag (length *B* and height *H*). The influence of the soil strength in the bag on the additional cohesion *c_T_* is indirectly reflected by the passive earth pressure coefficient *K*_p_. Therefore, even if the soil strength in the bag is low, the cohesion *c_T_* can still reach a larger value by adjusting the size of the soilbag or increasing the bag’s tensile force, because of which the soilbag can always have sufficient strength. Therefore, the contained material in the soilbag is not strictly limited. Theoretically, it can be all kinds of on-site excavation soil, construction waste, and even muddy soil.

Besides, several layers of soilbags form a hard shell layer on the surface of soft foundation [40]. Therefore, its strength and stiffness are greater than that of the soft soil layer under it. Meanwhile, it has an obvious diffusion effect on the stress generated by the upper load, which in return improves the bearing capacity of the foundation and reduces the settlement deformation.

### 2.3. Field Tests of Soilbag-Reinforced Foundations

#### 2.3.1. Test Site Conditions

In order to verify the feasibility of soilbags in foundation reinforcement, two test sites, the T5 test site with relatively good geological conditions and the T34 test site with poor geological conditions, were selected for field tests. Among them, the T5 hoisting site was located in the Lixia River shallow depression plain, which was originally cultivated land. Thus, the base soil conditions of this test site were relatively good, but there were still problems of high groundwater levels and serious water seepage. Table 1 presents the stratum condition and corresponding mechanical properties of the T5 test site. Meanwhile, the T34 test site was originally a pond with a high water content of sludge, and the surface layer was backfilled with plain soil of 60 cm thickness. The muddy soil excavated at the T34 site was “jelly-like”, with low permeability and low strength, and was prone to lateral sliding after oscillation or disturbance. The mechanical properties of the strata at the T34 test site are given in Table 2.

From the tables above, the characteristic values of the bearing capacity for each earth layer under the wind turbine hoisting platform were lower than the required foundation bearing capacity (*f_ak_* = 250 kPa), especially those of the plain fill and muddy soil layer close to the surface of the site which were even less than 100 kPa, far from the foundation bearing capacity requirements of this wind turbine hoisting platform site.

#### 2.3.2. Soilbag Design

Theoretically, the smaller the soilbag is, the higher its strength is. However, the bagging efficiency is lower. Comprehensively considering the earth conditions and construction efficiency of the test sites, the proposed dimensions of soilbags are 2.0 m × 1.0 m × 0.5 m (length × width × height) and each bag contains approximately 1.0 m^3^ of soil. The bag is made of polypropylene (PP) and has a mass per unit area of 230 g/m^2^. The properties measured of the PP bags are as follows: the warp and weft tensile strengths are 40 kN/m and 30 kN/m, respectively; the warp and weft elongations are both less than 18% [41], the strength retention rate of anti-ultraviolet aging (150 h irradiation with type II fluorescent UV lamp) is not less than 75% [42], and the color is black. In order to improve the tensile strength of the bag and facilitate hoisting and laying, the bag body is reinforced with belts which can also be used for hoisting.

The materials on-site that may be used for bagging are silt or plain soil mixed with brick slag. If the bag is filled with silt, the strength of the soilbag is calculated as 558.8 kPa according to Formulas (1) and (2) [9]; if the bag is filled with plain soil mixed with approximately 10% brick slag, its strength parameters obtained by direct shear tests on-site are that *φ* = 30°, *c* = 32 kPa, and the calculated value of soilbag strength is 614.9 kPa. The strength of the soilbag itself is greater than the ultimate bearing capacity of the hoisting platform of 500 kPa, so soilbag reinforcement can be an alternative method of replacing brick slag to improve the foundation bearing capacity of the on-site hoisting platform.

#### 2.3.3. Soilbag Layout

1. T5 test site

**Figure 2:**
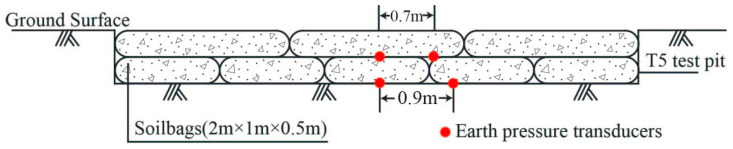
Schematic diagram of soilbags and earth pressure transducers layout for S1.

The test was carried out in a test pit with a plane size of 7 m × 7 m and a depth of 0.8 m. There was 2.2 m of thick plain fill on the surface of the T5 test site and 6.3 m thick medium compressible clay mixed with silty clay under it. According to previous research [29], the bearing capacity of foundations was found to increase with an increasing number of soilbag reinforcement layers, so the number of soilbag layers depends on the foundation condition. Due to the relatively good foundation condition of the T5 site, the field test scheme S1 was purposed to reinforce the foundation with two layers of soilbags (*n* = 2). Meanwhile, in order to discuss the influence of contained material in a bag on the reinforcement effect, 2 tests at the T5 sites were performed. The soilbags used in test S1a were filled with excavated plain soil mixed with approximately 10% of brick slag, and those in test S1b were filled with pure plain soil. The soilbag layers were staggered, and a certain number of earth pressure transducers were arranged on the base surface of the test pit and between the soilbags, as shown in Figure 2.

**Figure 3:**
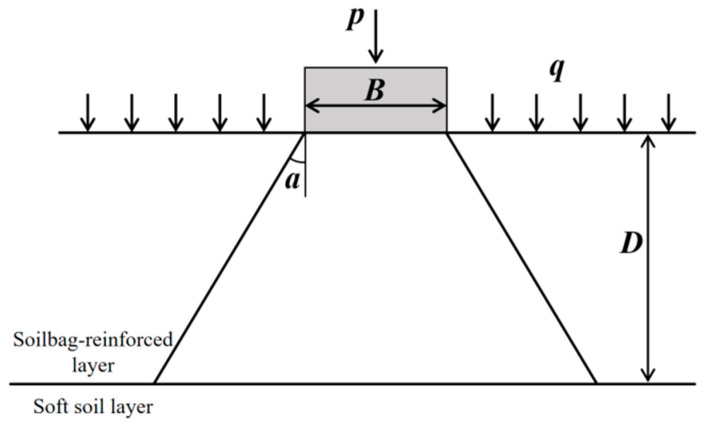
Schematic diagram of stress diffusion method.

It is assumed that the soilbag treatment layer is considered a reinforced soil cushion, with reference to the Technical Code for Ground Treatment of Buildings (JGJ 79–2012) [38]. Furthermore, the characteristic value of foundation bearing capacity can be estimated by the stress diffusion angle method [43] (as shown in Figure 3) based on the double-layer foundation theory [44].

The relationship between the ultimate bearing capacity of reinforced cushion foundation *f_u_*’ and the ultimate bearing capacity of soft soil foundation *f_u_* is as follows [43]:

(3) $$\begin{array}{c} f_{u}^{’}=f_{u}\left[1+2\left(\frac{D}{B}\right)\tan\alpha \right]\; \end{array}$$

Among

(4) $$\begin{array}{c} f_{u}=cN_{c}+qN_{q}\;\; \end{array}$$

(5) $$\begin{array}{c} N_{c}=\left(N_{q}-1\right)\cot\varphi \end{array}$$

(6) $$\begin{array}{c} N_{q}=\tan^{2}\left(\frac{\pi }{4}+\frac{\varphi }{2}\right)e^{\pi \tan\varphi }\;\; \end{array}$$

where *D* is the thickness of the reinforcement layer; *B* is the width of the strip foundation; *N_c_* and *N_q_* are the bearing capacity coefficients, which are related to the internal friction angle of soil; *φ* and *c* are the internal friction angle and cohesion of soil; *q* is the overload on both sides of the foundation; α is the stress diffusion angle; *p* is the load on strip foundation. Therefore, the value of the stress diffusion angle is the key to the calculation accuracy of the stress diffusion method. Furthermore, the value of the stress diffusion angle can generally be obtained through experiments. According to previous experimental research on soilbag technology, the stress diffusion angle is supposed to be approximately 40°.

The allowable bearing capacity *f_ak_* (also known as the characteristic value) of the foundation is generally half of the ultimate bearing capacity *f_u_*. According to article 5.2.4 of the Code for Design of Building Foundation (GB 50007-2011) [45], when the foundation width is greater than 3 m or the embedded depth is greater than 0.5 m, the characteristic value of foundation bearing capacity determined from the load test or other in-situ tests and empirical value method should be corrected using the following formula [45]:

(7) $$\begin{array}{c} f_{a}=f_{ak}+\eta_{b}\gamma \left(B-3\right)+\eta_{d}\gamma_{m}\left(D-0.5\right)\;\; \end{array}$$

where *f_a_* is the corrected characteristic value of foundation bearing capacity; *η_b_* and *η_d_* are the correction coefficients of foundation bearing capacity for foundation width and buried depth, respectively, which are taken by checking the table for values according to the category of soil under the foundation, thus for sludge soil *η_b_* is 0 and *η_d_* is 1.0; *γ* is the gravity of the soil under the foundation; *γ_m_* is the weighted average gravity of soil above the foundation ground.

For the T5 test pit, according to the soil mechanic parameters in Table 1, the average cohesion of the plain soil layer is 32 kPa, and the internal friction angle is 7.5°. According to Formulas (6) and (5), the bearing capacity coefficients calculated for soft soil are *N_q_* is 2.01, and *N_c_* is 7.48, and the correction coefficients of foundation bearing capacity are that *η_b_* is 0 and *η_d_* is 1.0 by checking the Code [45] for values. The thickness of the two-layer soilbags after compaction is assumed to be 0.8 m according to on-site measurements, so the calculation shows that the bearing capacity of the soilbag-reinforced foundation is about 259.48 kPa, which meets the design requirement of 250 kPa.

2. T34 test site

The tests at the T34 test site were conducted in 2 test pits with a plane size of 7 m × 7 m and a depth of about 1.5 m for each pit. The test pits were originally filled with high water content sludge. The surface layer was backfilled with about 60 cm of plain soil. The sludge layer was about 3 m thick and ‘jelly-like’ with low permeability and strength after site excavation, which was prone to lateral sliding after oscillation or disturbance. According to the foundation condition of the T34 test site, 2 reinforcement schemes, S2 and S3, were designed.

- Scheme S2: 5 layers of staggered soilbags (*n* = 5)

**Figure 4:**
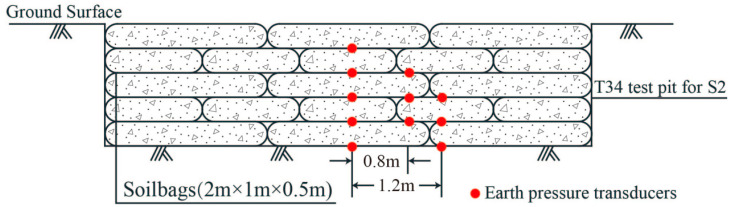
Schematic diagram of soilbags and earth pressure transducers layout for S2.

For the T34 site with sludge soil foundation, five layers of soilbags (*n* = 5) were arranged in a staggered manner. The soilbags were filled with excavated plain soil mixed with brick slag, and their layout is shown in Figure 4. Meanwhile, a certain number of earth pressure transducers were arranged on the base surface of the test pit and between each layer of soilbags. The thickness of the five-layer soilbags before rolling was 2.5 m, assuming that the thickness after rolling was 2.4 m. After calculations according to Formulas (3) to (7) by using the mechanical parameters of the strata in Table 2, the characteristic value of the foundation bearing capacity after soilbag reinforcement was about 282.8 kPa, which also meets the design requirement of 250 kPa.

- Scheme S3: 3 layers of staggered soilbags (*n* = 3) + bottom sludge treatment

**Figure 5:**
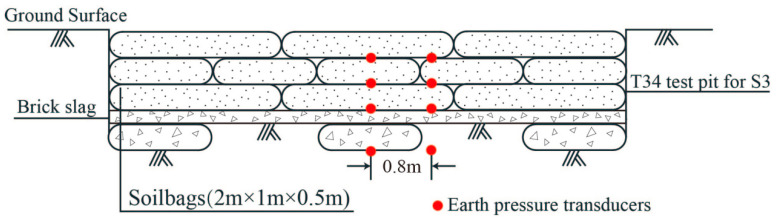
Schematic diagram of soilbags and earth pressure transducers layout for S3.

In this case, the permeable treatment of the bottom sludge was applied on the soft soil base first. Next, some permeable soilbags filled with brick slag were pressed into the excavated sludge soil to simulate riprap, then 0.1 m thick brick slag was filled and paved on the permeable soilbags to form a permeable cushion. As shown in Figure 5, once the bottom sludge treatment was completed, three layers of soilbags filled with silt near the site were arranged in a staggered manner. A certain number of earth pressure transducers were arranged on the base surface of the test pit and between each layer of soilbags.

#### 2.3.4. Field Test Overview

**Figure 6:**
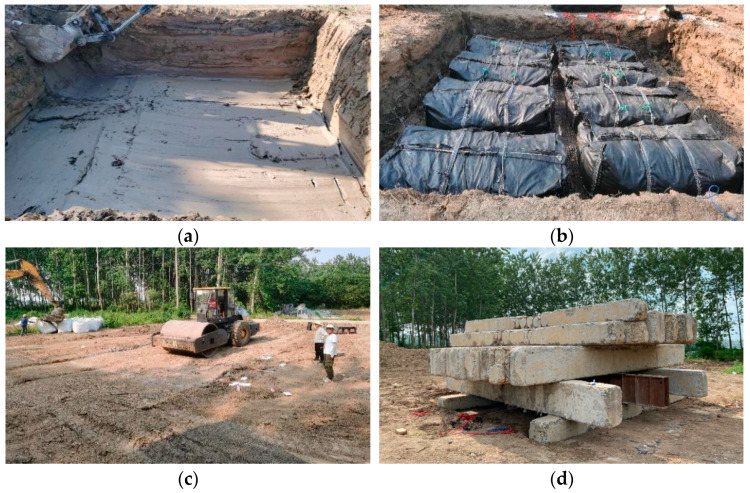
Construction procedure of foundation reinforcement with soilbags. (**a**) Excavation of test pits, (**b**) Centralized laying of soilbags, (**c**) Compaction by vibratory roller, (**d**) Plate load tests.

Figure 6 shows the site construction process of soilbags. First, the sludge was leveled after the excavation of the test pit, and then soilbags were directly laid on the sludge soil layer. Considering the difficulties of filling and loading soilbags in-situ on muddy soil manually or mechanically, the soilbags were bagged and loaded in advance at the selected soil stacking site, then hoisted to the test pits for centralized laying. In order to give full play to the tension effect of soilbags, the soilbags were laid in a crisscross arrangement between layers [38]. While each layer of soilbags was laid, the surface was beaten and leveled by an excavator. Finally, the test pits were filled and leveled up with plain soil once all soilbags were laid. Thereafter, a 20 t vibrating roller was used to roll over the surface of the test pits.

**Figure 7:**
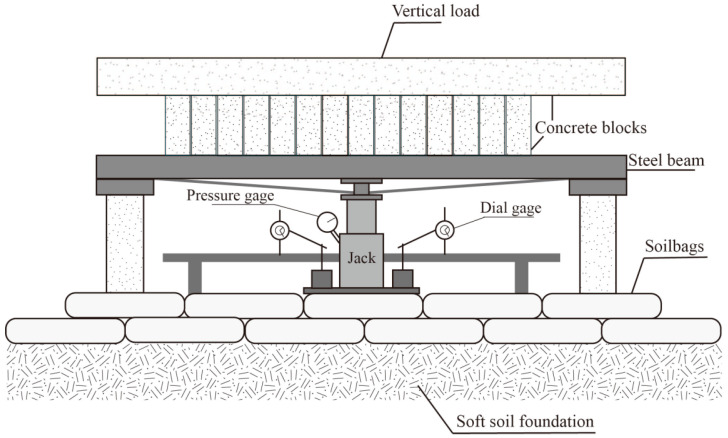
Schematic diagram of plate load tests.

The plate load tests based on the Code for Design of Building Foundation (GB 50007-2011) [45] were performed on soilbag-reinforced foundations after compaction, as shown in Figure 6d. The load was applied through a system comprising a hydraulic jack, a reaction frame and a loading plate, as shown in Figure 7. The loading plate was a square steel plate with a side length of 100 cm and a thickness of 2 cm. The back pressure was provided by the precast concrete blocks, whose basic size was 40 cm × 60 cm × 6 m. The main beam applied by back pressure was a 6 m long channel steel. In addition, 3 dial gages with divisions of 0.01 to 50 mm travel were used for settlement measurements. The gages were fixed to a reference beam and supported on external rods. The tests of S1 were applied in 7 loading stages of increments, and that of S2 and S3 were in 9 loading stages. For each load increment, settlements were measured at the following fixed times: zero, 10 min, 20 min, 30 min, 45 min, 60 min, and then every half an hour. When the settlement per hour was less than 0.1 mm within two consecutive hours, it was considered to be stable, and the next loading increment could be applied. The earth pressure between layers of soilbags was measured at different stages of the load tests.

**Figure 8:**
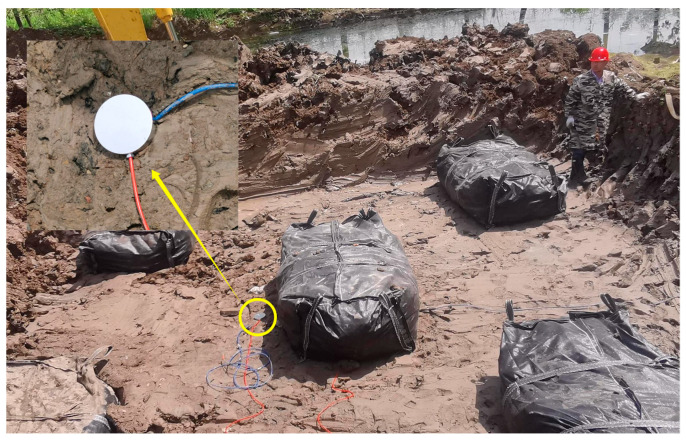
Installation of earth pressure transducers on-site.

During plate load tests, the earth pressure was measured by the JY-2020 vibrating wire earth pressure transducers of Jinyan Engineering Instrument Co., Ltd. in Yixing, China, with a range of 0~600 kPa. Figure 8 shows the installation of the earth pressure transducers. The earth pressure transducers were placed horizontally at the designed points. The wirings of the transducers were pulled out of the test pit to facilitate data measurement.

## 3. Results and Analysis

### 3.1. p-s Curve

#### 3.1.1. S1

**Figure 9:**
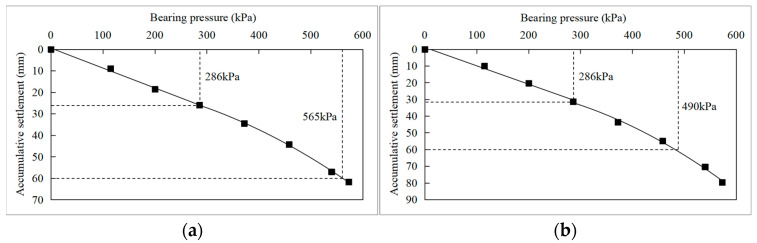
p-s curves of plate load tests for S1 at T5 test site. (**a**) S1a, (**b**) S1b.

Table 3 shows the cumulative settlement corresponding to the loading of each stage in the plate load tests of the S1 scheme (*n* = 2) at the T5 site. Taking the accumulative settlement s as the horizontal axis and the bearing pressure p as the vertical axis, the p-s curves of S1a and S1b are drawn as shown in Figure 9.

It can be seen from the results that the settlement changed linearly when the load was less than 286 kPa. Thus the proportional limit can be taken as 285 kPa for test S1a, in which the soilbags used for foundation reinforcement were filled with plain soil mixed with brick slag. The ultimate load was determined as the vertical load while the ratio of settlement to bearing plate diameter was 0.06, i.e., the cumulative settlement was 60 mm in this case; hence the ultimate load of test S1a was 565 kPa. According to the regulations on eigenvalue values in the Technical Code [38]: if the p-s curve has a proportional limit, the load value corresponding to the proportional limit would be taken as the characteristic value of bearing capacity; if the ultimate load is less than two times the corresponding proportional limit, half of the ultimate load is taken as the characteristic value of bearing capacity. In test S1a, the ultimate load was less than twice the proportional limit, so the characteristic value of bearing capacity was taken as half of the ultimate load value, which was 282.5 kPa. This result meets the design requirement of 250 kPa, which is close to the characteristic value of bearing capacity calculated by considering the soilbag treatment layer as a reinforced soil cushion.

For S1b, the foundation was reinforced by soilbags filled with plain fill. During the loading process, the settlement of the test point changed linearly in the early loading stage so that the proportional limit could be taken as 285 kPa, similar to test S1a. The vertical load corresponding to the cumulative settlement of 60 mm was about 490 kPa, which was determined as the ultimate load. Thus, the characteristic value of bearing capacity for S2 should be taken as half of the ultimate load value, which was 245 kPa, slightly less than the design requirement of 250 kPa, basically meeting the design requirements.

The test results of S1a and S1b indicated that, for the foundation of clay mixed with silt clay, two layers of soilbags could improve the bearing capacity of the soft foundation. It was found that both soilbags with excavated plain fill and those with plain fill mixed with brick slag as the contained material can increase the bearing capacity of soft foundations and reduce settlement under external loads. The characteristic value of bearing capacity obtained from the tests of the two infilled materials met the hoisting requirements. This validates that the contained material in soilbags is not strictly limited. Nevertheless, comparing the reinforcement effect of S1a and S1b, the bearing capacity obtained by S1a, which used the excavated plain fill mixed with brick slag, was higher than that of S1b, which used the pure plain soil in the bag. Furthermore, the footing settlement of S1a was less than that of S1b under vertical stress. It was found from the comparison of S1a and S1b that the reinforcement effect of filling plain soil is not as good as that of filling plain soil with brick slag. Hence, plain soil mixed with brick slag is the preferred contained material in this case. It is recommended to increase the number of layers of soilbags, for example, to three layers, with the use of pure plain soil as the soilbag filling material to ensure the same effect as S1a.

There are two factors to produce such a result:

(1) The compressibility of soilbags filled with pure plain soil was greater, so the soilbag compression deformation of S1b was greater under the same external loads, which increased the footing settlement during plate load tests.
(2) After construction compaction, the thickness of the soilbag filled with plain soil was about 0.3–0.35 m, which was slightly less than that of the soilbag containing plain soil mixed with brick slag, which was 0.35–0.4 m. Thus, the total thickness of the reinforcement layer of S1b was slightly less than that of S1a; in this way, the additional stress transmitted to the soft soil under the soilbag reinforcement layer was greater. Therefore, the reinforcement effect of soilbags infilled with plain soil mixed with brick slag was better than with pure plain soil.

The results of field tests confirmed that using soilbag technology to reinforce the foundations of T5 is feasible. Therefore, the proposed foundation reinforcement scheme for the hoisting platform of T5 is two layers of soilbags infilled with plain soil mixed with slag brick or three layers of soilbags infilled with pure plain fill if it is not convenient to obtain brick slag on-site.

#### 3.1.2. S2 and S3

**Figure 10:**
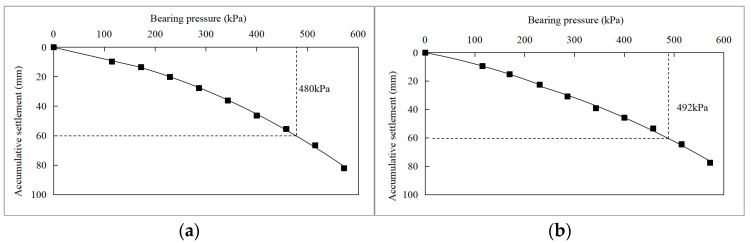
p-s curves of plate load tests for S2 and S3 at T34 test site. (**a**) S2, (**b**) S3.

Table 4 shows the cumulative settlement corresponding to each stage of loads in the plate load tests of the “5-layer soilbags” test scheme S2 and the “3-layer soilbags + bottom sludge treatment” test scheme S3 at the T34 site. Taking the cumulative settlement s as the horizontal axis and the bearing pressure p as the vertical axis, the p-s curves of S2 and S3 are shown in Figure 10.

It can be seen from the results that the footing settlement increases gradually with the increase in the applied load, and there is no obvious turning point in the p-s curve. According to specifications [38,45], the vertical loading was approximately 480 kPa, while the settlement was 60 mm, which was the ultimate load in test S2 with five layers of soilbags. Therefore, the characteristic value of bearing capacity was taken as half of the ultimate load, which was 240 kPa, slightly less than the design requirement of 250 kPa. However, the footing settlement increased slowly until the last stage of loading was applied. It was observed that the foundation was stable and did not reach the ultimate failure state during plate load tests. Similarly, the ultimate load corresponding to the settlement of 1/6 load plate diameter of S3 was 492 kPa. Thus the characteristic value of the bearing capacity of S3 was 247.5 kPa. Furthermore, it was found that the bearing capacity of sludge foundations has been improved by the permeable treatment of the sludge bottom; even the foundation was reinforced by fewer layers of soilbags. Therefore, the experimental results of the two schemes, S2 and S3, meet the requirements of the foundation-bearing capacity of the hoisting platform.

Therefore, for foundations with poor conditions, such as the deep sludge soil base at the T34 sites, the soilbag reinforcement can effectively increase the bearing capacity of foundations. Nevertheless, more layers of soilbags were needed compared with S1 at the T5 site. The results of the two schemes, S2 and S3 were similar. The reinforcement cushion of five-layer soilbags and three-layer soilbags while the sludge surface is permeably treated can increase the foundation bearing capacity to the design requirements. Based on the technical principle of soilbags, the role of soilbags in reinforcing the foundation can be considered from the following two aspects: on the one hand, the soilbags formed an excellent skeleton effect by restraining the soil through the bags during the load tests, which increased soil strength, on the other hand, the channels for drainage and consolidation were formed between the bags, which was beneficial to the drainage and consolidation of the foundation. Furthermore, due to the relatively high permeability of bottom sludge after permeable treatment, the drainage efficiency can be accelerated. That is why the same reinforcement effect can be achieved even if the number of bags was reduced to three layers in S3.

The results of field tests S2 and S3 indicated that more layers of soilbags are needed for soft foundations with poor foundation conditions. Soilbag technology combined with permeable treatment of bottom sludge is an effective foundation reinforcement method for deep silt soil bases. The number of soilbag layers could be reduced by pressing in a permeable soilbag layer under the same soft soil foundation conditions.

### 3.2. Earth Pressure Distribution

**Figure 11:**
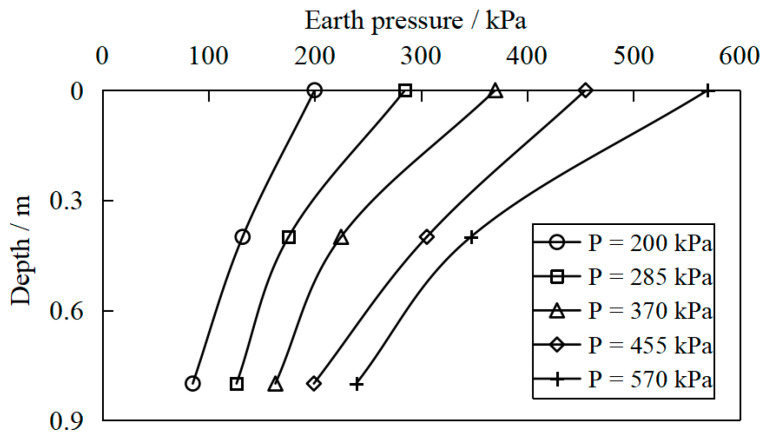
Variation in earth pressure along the depth below the center point of the bearing plate for S1a.

**Figure 12:**
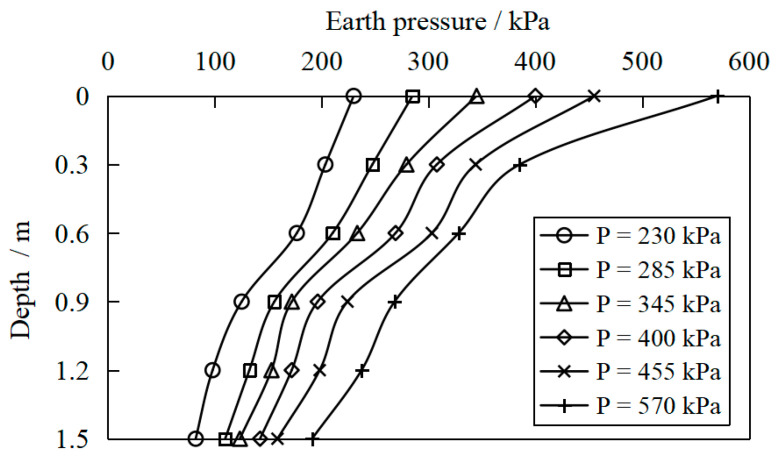
Variation in earth pressure along the depth below the center point of the bearing plate for S2.

**Figure 13:**
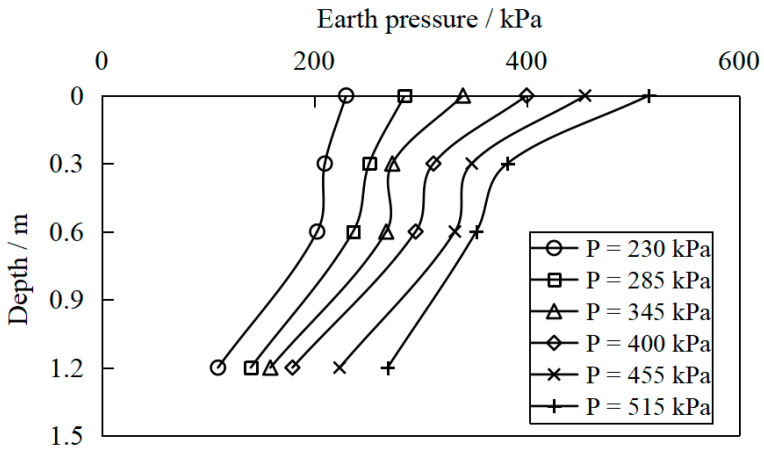
Variation in earth pressure along the depth below the center point of the bearing plate for S3.

Figure 11, Figure 12 and Figure 13 show the variation of earth pressure along the depth below the center point of the pressure-bearing plate measured for S1a, S2 and S3, respectively. It was found that the earth’s pressure gradually decreased with the depth. However, the earth pressure on the surface of the site was the largest, indicating that the vertical load diffused in the soilbag reinforcement cushion. Nevertheless, with the increase in vertical pressure, the exertion of soilbag tension is more obvious, the stiffness of the reinforcement layer increases, and the attenuation of soil pressure with depth becomes more significant.

**Figure 14:**
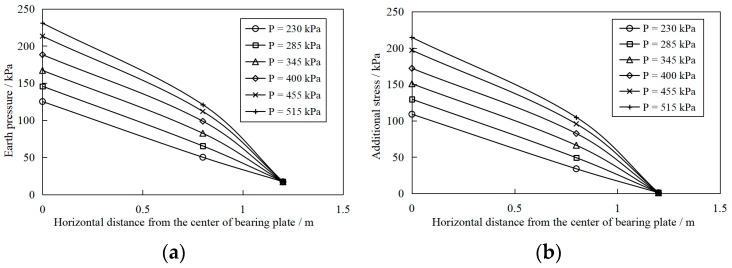
Variation in earth pressure and additional stress at a depth of 0.9 m with horizontal distance from the center of the bearing plate. (**a**) Variation in earth pressure, (**b**) Variation in additional stress.

Figure 14a shows the horizontal distribution of soil pressure measured at a depth of 0.9 m in the S2 test scheme. It was observed from the results that under the same depth and the same vertical load, the earth pressure under the center of the bearing plate was the largest. With the increase in the horizontal distance from the center of the bearing plate, the earth pressure gradually decreased. Besides, the earth pressure measured under different vertical loads at the measuring point 1.2 m horizontally from the plate center was almost the same. According to the research, the earth pressure measured includes two parts; the self-weight stress of the upper soil and the additional stress caused by vertical loads. The specific gravity of the soilbag reinforcement layer is considered 18 kN/m^3^, so the self-weight stress at a depth of 0.9 m is 16.2 kPa. The earth pressure after deducting the self-weight stress is the additional stress caused by the vertical load, and its distribution is shown in Figure 14b. It can be seen that the additional stress values under different loads at the measuring point, which is 1.2 m away from the pressure-bearing plate center, are 0 kPa. Therefore, the stress diffusion angle of the soilbag reinforcement layer is approximately 38° based on the stress diffusion angle method [43], which has excellent agreement with the assumed value of foundation bearing capacity calculation by the stress diffusion angle method.

## 4. Discussion

The conventional approach for reinforcing foundations in wind farms’ hoisting platforms involves excavating soft soil and substituting it with construction waste. Utilizing soilbags as sustainable construction material to enhance soft foundations offers several advantages over this conventional method, as it effectively makes full use of the available solid wastes on-site, reducing construction costs and facilitating the easy restoration of the original foundation post-construction.

The findings from plate load tests conducted on soft foundations reinforced by soilbags with solid wastes have substantiated that soilbag reinforcement significantly improves the bearing capacity of soft foundations under vertical loading conditions. Comparing the test results from this study with those of Xu [34] and Wang [29] in other projects, the specific details of each test are presented in Table 5.

The results from the three aforementioned studies indicate that soilbag reinforcement can enhance foundation-bearing capacity by 2–5 times compared to unreinforced foundations. However, the extent of improvement varies and is largely dependent on foundation conditions, the number of soilbag layers, soilbag dimensions, and construction methods. By comparing the results between test 4 and test 5, as well as between test 6 and test 7, it is evident that the bearing capacity increases with an increasing number of soilbag reinforcement layers under the same original foundation conditions. This observation also supports the findings of test 2 and test 3, indicating that for soft foundations with poor conditions such as S2, more soilbag layers are needed to achieve a similar reinforcement effect. Additionally, soilbags filled with different infill materials, such as plain fill, sand and sandy clay, exhibit excellent reinforcement effects. This outcome can be attributed to soilbags reinforcing foundations by utilizing the tension of the bag to restrain the soil. Thus, the bearing capacity of the reinforced foundation is not significantly affected by the material in the bag. Therefore, soilbag reinforcement enables efficient utilization of excavated solid waste, offering advantages of high construction efficiency, easy reclamation, low cost, and good environmental protection compared to the conventional excavation and replacement method.

This study investigates the application of soilbag technology in reinforcing soft soil foundations through on-site plate load tests. The experimental results can serve as a basis for similar projects involving the treatment of soft foundations. However, it is important to note that certain influencing factors were not considered due to limitations in construction costs and on-site conditions. Future experimental research can delve deeper into exploring factors such as the arrangement of soilbags and the combination of soilbag reinforcement with drainage boards.

## 5. Conclusions

This paper investigates the feasibility of soilbag material in the soft foundation treatment based on the construction of a wind farm in Jiangsu Province, China. The research comprises theoretical analysis and field tests, leading to the following key findings:

(1) Soilbags possess high strength due to the additional cohesion produced by the bag tension under external force. The feasibility of soilbag reinforcement technology in the soft foundation treatment is confirmed through plate load tests. Soilbag reinforcement using solid waste increases the bearing capacity of soft foundations and reduces settlement under external loads. Different solid residues can be used as the material contained in the soilbag, including excavated soil and brick slag residues, while the soilbag with plain fill mixed with brick slag has a better reinforcement effect than that with pure plain fill.
(2) Soilbag technology combined with permeable treatment of bottom sludge is an effective foundation reinforcement method for deep silt soil bases. Furthermore, the number of soilbag layers required for the same reinforcement effect can be reduced by pressing a permeable soilbag layer in the bottom sludge under the same foundation conditions.
(3) The earth pressure decreases gradually with depth, indicating that the vertical load diffuses in the soilbag cushion. As the vertical load increases, the exertion of soilbag tension becomes more significant, the stiffness of the reinforcement layer increases, and the attenuation of soil pressure with depth becomes more pronounced. The earth pressure under the bearing plate is the largest at the same depth, and the earth pressure decreases with the horizontal distance from the center of the bearing plate. The stress diffusion angle of the soilbag reinforcement layer obtained from the tests is approximately 38°.
(4) Compared to the conventional excavation and replacement approach, the soilbag reinforcement material can meet the construction requirements while offering many advantages, such as cost savings, a shorter construction period, high construction efficiency, and easy reclamation. In addition, soilbags infilled with solid wastes make full use of local materials while also being environmentally friendly. Therefore, soilbag technology has promising prospects for application in the construction of hoisting platforms for plain wind farms near lakes.

## Figures and Tables

**Table 1:** Mechanical properties of the strata at T5 test site.

Stratum No.	Stratum Name	Thickness (m)	SPT Blow Count	Characteristic Value of Bearing Capacity (kPa)	Cohesion (kPa)	Angle of Internal Friction (°)
①	Plain fill	2.2	~	60	32	7.5
③	Clay mixed with silty clay	6.3	8~12	180	62.6	11.7
③−1	Silt	3.6	15~17	170	3.1	32.1
④	Clay mixed with silty clay	9	10~12	240	90.1	14.5
④−1	Silt	2.1	16	190	5.8	30.3
⑤	Silty clay	3.8	9~10	180	54.3	10.8

**Table 2:** Mechanical properties of the strata at T34 test site.

Stratum No.	Stratum Name	Thickness (m)	SPT Blow Count	Characteristic Value of Bearing Capacity (kPa)	Cohesion (kPa)	Angle of Internal Friction (°)
①	Plain fill	0.6	~	60	32.0	7.5
②	Muddy soil	2.8	1	50	7.6	1.7
③	Clay mixed with silty clay	8.6	8~13	180	62.6	11.7
④−1	Silt	4.0	17~22	190	5.8	30.3
④	Clay mixed with silty clay	7.6	11~14	240	90.1	14.5
⑤	Silty clay	5.7	10~12	180	54.3	10.8

**Table 3:** Accumulated settlement of plate load tests at T5 test site.

Loads p at Each Stage (kPa)		115	200	285	370	455	540	570
Accumulative settlement s (mm)	S1a	8.94	18.63	26.02	34.44	44.27	57.04	61.69
	S1b	10.09	20.32	31.44	43.67	54.88	70.33	79.68

**Table 4:** Accumulated settlement of plate load tests at T34 test site.

Loads p at Each Stage (kPa)		115	170	230	285	345	400	455	515	570
Accumulative settlement s (mm)	S2	9.65	13.45	20.12	27.78	36.11	46.23	55.49	66.44	81.98
	S3	9.42	15.21	22.56	30.67	39.12	45.82	53.43	64.32	77.77

**Table 5:** Bearing capacity of soilbag-reinforced foundations from other research.

No.	Resource	Bearing Capacity/kPa		Number of Soilbag Layers	Dimension of Soilbags	Infill Material for Soilbags
		Unreinforced Foundation	Soilbag-Reinforced Foundation			
1	S1a	60	245	2	2 m × 1 m × 0.5 m	excavated plain fill
2	S1b	60	282.5	2		plain fill with brick slag
3	S2	50	240	5		plain fill with brick slag
4	Xu, 2008	70	160	2	0.5 m × 0.5 m × 0.1 m	sand
5		70	240	3		sand
6	Wang, 2019	96	135	2	0.4 m × 0.4 m × 0.1 m	excavated sandy clay
7		96	170	4		excavated sandy clay

## Data Availability

Not applicable.
